# Impact evaluation of a community-based intervention for prevention of cardiovascular diseases in the slums of Nairobi: the SCALE-UP study

**DOI:** 10.3402/gha.v9.30922

**Published:** 2016-03-24

**Authors:** Steven van de Vijver, Samuel Oji Oti, Gabriela B. Gomez, Charles Agyemang, Thaddaeus Egondi, Eric Moll van Charante, Lizzy M. Brewster, Catherine Hankins, Zlata Tanovic, Alex Ezeh, Catherine Kyobutungi, Karien Stronks

**Affiliations:** 1 African Population and Health Research Center, Nairobi, Kenya; 2 Department of Global Health, Academic Medical Center, University of Amsterdam and Amsterdam Institute for Global Health and Development, Amsterdam, The Netherlands; 3 Department of Global Health and Development, London School of Hygiene and Tropical Medicine, London, UK; 4 Department of Public Health, Academic Medical Center, University of Amsterdam, Amsterdam, The Netherlands; 5 Department of Family Medicine, Academic Medical Center, University of Amsterdam, Amsterdam, The Netherlands; 6 Departments of Vascular and Internal Medicine, Academic Medical Center, University of Amsterdam, Amsterdam, The Netherlands; 7 Department of Infectious Disease Epidemiology, London School of Hygiene and Tropical Medicine, London, UK; 8 Amsterdam Institute for International Development (AIID), Faculty of Economics and Business Administration, Vrije Universiteit Amsterdam, Amsterdam, The Netherlands

**Keywords:** cardiovascular disease, prevention, awareness, treatment, hypertension, blood pressure, slum, urban poor, Kenya

## Abstract

**Background:**

A combination of increasing urbanization, behaviour change, and lack of health services in slums put the urban poor specifically at risk of cardiovascular disease (CVD). This study aimed to evaluate the impact of a community-based CVD prevention intervention on blood pressure (BP) and other CVD risk factors in a slum setting in Nairobi, Kenya.

**Design:**

Prospective intervention study includes awareness campaigns, household visits for screening, and referral and treatment of people with hypertension. The primary outcome was overall change in mean systolic blood pressure (SBP), while secondary outcomes were changes in awareness of hypertension and other CVD risk factors. We evaluated the intervention's impact through consecutive cross-sectional surveys at baseline and after 18 months, comparing outcomes of intervention and control group, through a difference-in-difference method.

**Results:**

We screened 1,531 and 1,233 participants in the intervention and control sites. We observed a significant reduction in mean SBP when comparing before and after measurements in both intervention and control groups, −2.75 mmHg (95% CI −4.33 to −1.18, *p=*0.001) and −1.67 mmHg (95% CI −3.17 to −0.17, *p=*0.029), respectively. Among people with hypertension at baseline, SBP was reduced by −14.82 mmHg (95% CI −18.04 to −11.61, *p*<0.001) in the intervention and −14.05 (95% CI −17.71 to −10.38, *p*<0.001) at the control site. However, comparing these two groups, we found no difference in changes in mean SBP or hypertension prevalence.

**Conclusions:**

We found significant declines in SBP over time in both intervention and control groups. However, we found no additional effect of a community-based intervention involving awareness campaigns, screening, referral, and treatment. Possible explanations include the beneficial effect of baseline measurements in the control group on behaviour and related BP levels, and the limited success of treatment and suboptimal adherence in the intervention group.

## Paper context

Cardiovascular disease is reaching epidemic levels among the urban poor in SSA, with hypertension being the major risk factor. This study is the first evaluation of an intervention to reduce cardiovascular risk in these settings, while focusing on awareness, screening, treatment, and adherence of hypertension. The paper shows that reduction of blood pressure can be reached in these settings; however, it is important to determine in future studies what the key drivers behind this are.

## Background

Cardiovascular disease (CVD) burden is rapidly rising in sub-Saharan Africa (SSA), with the absolute number of annual deaths increasing from 1.0 million in 2000 to 1.3 million in 2008 and expected to increase to approximately 2.1 million deaths annually by 2030 (1). CVD deaths occur in African adults on average 10 or more years earlier than among adults in Europe and North America (2). Young adults in SSA who are in the prime of their economically productive years are at a higher risk of developing CVD and dying prematurely compared with their western counterparts, posing a serious threat to the economies of countries in SSA (3).

Hypertension is the main risk factor for CVD and has recently become the leading risk factor for death in SSA (4). Between 1990 and 2010, mean systolic blood pressure (SBP) in Kenya rose from approximately 125 to around 130 mmHg (5). Over the same period in Europe and North America, average blood pressure (BP) decreased by approximately 3 mmHg (5). This is partly explained by the fact that Kenya and other countries in SSA are in an earlier phase of the epidemiological transition (6) from an environment with predominantly nutritional deficiencies and infectious diseases to one with degenerative diseases, such as CVD related to industrialization, urbanization, and changes in behaviour. Earlier studies in the Kenyan context have shown how urban transition increases BP among the population within a brief period of time (7), which is possibly due to dietary changes, with increased sodium, fat, and sugar, and decreased physical exercise. In addition, urban transition and life in the slums are linked to increased psychosocial stress, violence, and insecurity, which can lead to increased risk of CVD (8, 9).

Improving the availability of and access to appropriate medication for people with hypertension is critical in order to create a feasible and cost-effective way of slowing down rising trends of CVD mortality in SSA, where public health regulations and policies are weak (10–12). Countries in SSA are bearing a double burden of disease, with the rise of non-communicable diseases occurring against a backdrop of high prevalence of major communicable diseases such as HIV, tuberculosis, and malaria (13). In this context, health care structures are overburdened, specifically among the growing urban poor in SSA. Currently, it is estimated that between 60 and 70% of the urban population in SSA live in slums where reliable health care facilities are virtually non-existent (14). Kenyan slum dwellers are dependent on private health care facilities that are unregulated and often run by unqualified personnel who have very low understanding and awareness of the rising burden of CVD. They therefore rarely measure BP and assess the presence of other CVD risk factors (15), while medication for hypertension is often not available. Access to private health facilities in turn increases patients’ out-of-pocket expenditures, since 90% of the slum population do not have health insurance (16). The urban poor face a high prevalence of hypertension but have low levels of awareness, access to treatment, and BP control (17). Although policy documents have been developed addressing the CVD epidemic in low-income settings (10), to our knowledge, there have been no studies to evaluate their recommendations in SSA (18). This study aims to evaluate the impact of a community-based CVD prevention programme on BP and other CVD risk factors in a slum setting in Nairobi, Kenya.

## Methods

### Setting

The study was a prospective evaluation study of a community-based intervention among the urban poor, carried out in two slums called Korogocho and Viwandani. They each had around 35,000 residents at the time of the study. They are located about 5 to 10 km from the central business district of Nairobi, Kenya, with 8 km between them. High levels of poverty and unemployment, combined with a lack of social amenities, including limited access to quality primary health care and recreation facilities, characterize these slums. Opportunities for healthy lifestyle and medical support are few, increasing CVD risk. The intervention took place in Korogocho, with Viwandani designated as the control community. The control population had access to CVD standard of care, meaning that the only access to health care was actually outside the slum.

### Intervention

The multi-component intervention was designed based on earlier studies on CVD risk factors conducted by the African Population and Health Research Center (APHRC) within the same setting (17, 19, 20), review of available literature on effective community-based interventions for CVD prevention (11), and input from various stakeholders and experts from both the private sector and the public health care delivery system (21). The intervention has been described in more detail elsewhere (22). Briefly, the four components of the intervention were as follows:

1. Raising awareness prior to the door-to-door campaign: Through radio jingles on the local radio station Koch FM and awareness campaigns through visits at churches, mosques, and other public spaces within the slum to create understanding of CVD and increase participation in the program.
2. Improving access to screening: Through door-to-door household visits by community health workers (CHWs) who measured BP and other anthropometric outcomes and provided brief counselling on cardiovascular risk factors to all consenting adults aged 35 years and above. Because earlier research revealed that unhealthy diets and reduced physical exercise, aligned with the epidemiological transition taking place in these settings, increase CVD risk; the CHWs assessed study participants’ level of engagement in risky lifestyle behaviour, including tobacco use, alcohol use, physical activity levels, and dietary habits. Consequently, they provided brief counselling assistance (BCA) on healthy lifestyle modification using the six A's approach –Ask, Advice, Assist, Arrange, Agree, and Affirm (23). Traditional BCA does not include the sixth A (Affirm) as a separate entity. However, due to the importance of lifestyle change, CHWs encouraged study participants to continue with any healthy lifestyle behaviour in which they were currently engaged.
3. Facilitating access to treatment: Through distribution of vouchers for a free visit to the intervention clinic to persons identified with hypertension; health service improvements (nurses and clinical officers were trained, primary care guidelines for hypertension management developed, and equipment supplied); opening of a clinic at a central location in the slum, within walking distance for the local population, and also open on the weekends to increase access to care for daily labourers who work during weekdays; and incentives to CHWs to encourage people identified with hypertension to come to the clinic for an initial visit.
4. Promoting long-term retention in care: Through incentives to CHWs to encourage patients to visit the clinic during the first 6 months, medication subsidies, creation of patient support groups to build knowledge and understanding through train-the-trainer sessions, and SMS reminders to improve adherence.

### Evaluation

We evaluated the intervention's impact through two cross-sectional surveys conducted at baseline and after 18 months in the control and intervention communities. The evaluation took place within the area covered by the Nairobi Urban Health Demographic Surveillance System (NUHDSS) that has been run by the APHRC since 2002 (24). As the NUHDSS was under close supervision during the study period of 18 months, there were, to the best of our knowledge, no other related interventions coinciding with our study in both Korogocho and Viwandani.

The planned primary outcomes for this evaluation were the difference in the change in the proportion of the study population that were at moderate or high risk of CVD (>10% fatal and non-fatal) and the difference in the change in mean SBP both at population level and among people with hypertension at baseline. Due to budgetary constraints and an ongoing feasibility study (25), we decided during the study to abandon glucose measurements and limit the primary outcome to changes in SBP. Secondary outcomes included hypertension prevalence, change in mean diastolic blood pressure (DBP), the proportion of respondents who were aware of their hypertension, and the prevalence of other CVD risk factors in both intervention and control groups.

### Sample size and sampling

Participants in the surveys were adults aged 35 years or older who were living in the Korogocho and Viwandani slums and gave informed consent to participate in the study. Exclusion criteria included pregnancy, a history of CVD (myocardial infarction, stroke, heart failure, or angina pectoris), psychiatric illness, and inability to provide informed consent.

A total sample size of 3,220 respondents (1,610 per site) was needed in order to detect a 5% reduction at endline in the proportion of adults who are at moderate or high risk of CVD in the intervention population versus no change in the control population (assuming both populations have similar a baseline prevalence of 25%) as a primary outcome. The sample size took into account a 10% non-response rate, a power of 90%, and an alpha of 0.05 (22).

The NUHDSS database, which contains the names, locations, sex, and dates of birth of 15,000 individuals aged 35 years and above in both slums, was used as a sampling frame. Each individual in the NUHDSS has a unique identification number (ID). Subsequently, a list of random numbers was generated (26), and individuals with IDs matching the random numbers were selected to participate in the study.

### Data collection

Demographic and socio-economic data were collected during household visits by trained field interviewers. Questionnaires included detailed questions on lifestyle regarding CVD risk factors such as diet, physical activity, and tobacco and alcohol uptake. In the intervention site (Korogocho), trained CHWs collected the baseline study data. After conducting each interview, they started the counselling that was part of the intervention. In the control site (Viwandani), field interviewers collected the baseline study data. Field interviewers and CHWs were trained together. Anthropometric measures, including hip, waist, height, weight, and BP, were collected at study baseline in August 2012 and 18 months later in February 2014. BP was measured three times consecutively after 5 min of rest in the upper left arm with the arm positioned at heart level against the chest, using validated Digital Automatic Blood Pressure Monitors from OMRON^®^. We took the average of the last two BP measurements for analysis. Data quality was enforced by field supervisors who conducted frequent and random sit-in and spot-checks of interviews. These supervisors also performed office editing of completed questionnaires to check for completeness and consistency of collected data.

### Analysis

We compared intervention and control participants’ characteristics for both baseline and endline surveys using *t*-test for continuous variables and chi-square test for categorical variables. Changes in study outcomes were assessed in two cohorts of participants: all adults aged 35 years and older participating in both baseline and endline surveys, and those who were hypertensive at baseline and participated in the endline survey. The differences were evaluated using logistic and linear regression for binary and continuous outcomes, respectively. The analysis was adjusted for participant age, sex, education, and income. Assuming parallel trends within the two communities of Korogocho and Viwandani in the absence of any intervention, difference-in-difference estimates were used to assess the impact of the intervention on outcomes. An interaction term between group (intervention/control) and survey wave (baseline/endline) was included in each model. All the analyses were performed using STATA statistical software (26).

## Results

### Population characteristics

**Fig. 1:**
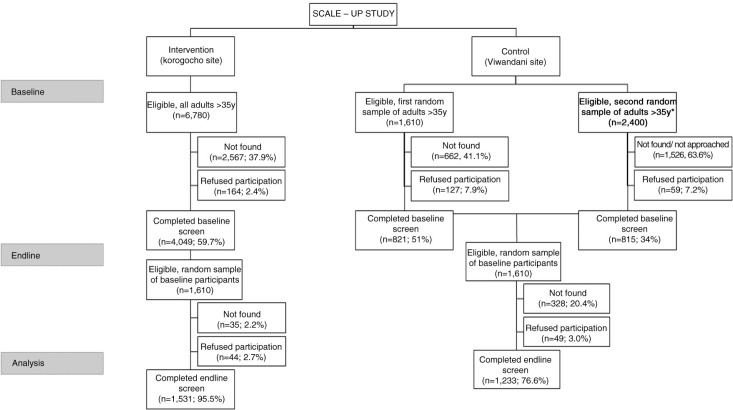
Overview of recruitment of the SCALE-Up study following CONSORT reporting.

We screened 1,531 and 1,233 participants in the intervention and control sites. During the baseline study, refusal rates at the intervention and control sites were 2.4 and 7.5%, respectively. Due to high population mobility, selected participants were difficult to find, so we had to resample a second group and stopped recruiting once we reached the target. The complete recruitment can be seen in Fig. 1 following CONSORT guidelines. Specifically, younger and male participants were more difficult to find in both intervention and control sites. As the distribution of our group was different from the expected distribution based on the NUHDSS database, we weighted the results. The prevalence of hypertension and mean SBP were similar between the sites at baseline, but the DBP was higher in the control group than in the intervention group (81.4 mm Hg vs. 83.0 mm Hg, *p=*0.001). The intervention group was older, less educated, and poorer. It was comprised of more females. It had less alcohol and tobacco use but more physical activity compared with the control group (Table 1).

**Table 1:** The distribution of the study population interviewed in both baseline and endline surveys (intervention, *n*=1,531; control, *n*=1,233)

	Baseline					Endline				
		
	Intervention		Control			Intervention		Control 1		
						
	*n*	%	*n*	%	*p*-value^0^	*n*	%	*n*	%	*p*-value^1^
Proportion with SBP≥140 mmHg and/or DBP≥90 mmHg	388	24.1	265	20.2	0.017	360	20.6	242	18.1	0.111
Mean systolic blood pressure	1,516	123.5	1,204	122.8	0.303	1,519	121.0	1,232	121.1	0.867
Mean diastolic blood pressure	1,516	81.4	1,204	83.0	0.001	1,519	80.8	1,232	81.9	0.009
Age										
30 to 40	286	22.8	442	41.4	0.000	195	24.0	287	31.6	0.000
41 to 50	611	40.1	566	41.3	0.541	592	38.8	665	47.8	0.000
51 to 60	368	23.5	171	13.4	0.000	437	23.7	213	15.8	0.000
Above 60	266	13.6	54	3.9	0.000	307	13.5	68	4.8	0.000
Sex										
Female	859	49.5	463	21.9	0.000	671	48.8	772	72.8	0.000
Male	672	50.5	770	78.1	0.000	860	51.2	461	27.2	0.000
Education										
No education	278	15.6	51	3.3	0.000	244	12.9	42	2.9	0.000
Primary incomplete	332	20.6	127	9.7	0.000	451	27.4	111	8.2	0.000
Primary complete	685	46.8	622	48.9	0.278	621	43.7	631	49.9	0.002
Secondary+	236	17.0	433	38.1	0.000	215	16.1	449	39.0	0.000
Income (Kshs.)										
<5,000	798	62.0	219	17.1	0.000	685	55.8	162	12.3	0.000
5,000–9,999	364	31.1	523	47.5	0.000	359	33.3	405	34.8	0.483
≥10,000	77	6.9	333	35.4	0.000	117	11.0	549	53.0	0.000
Other risk factors										
Tobacco use	159	11.6	192	19.2	0.000	187	14.2	149	13.8	0.793
Alcohol use	252	18.2	303	29.4	0.000	282	21.3	250	23.1	0.296
Insufficient fruit and vegetable consumption	1,073	70.5	878	71.3	0.660	1,114	73.1	966	79.1	0.001
Inadequate physical activity	75	4.2	27	1.7	0.000	75	4.3	97	7.3	0.001
Levels of awareness, treatment and control										
% of those with high BP aware of it	156	9.0	81	5.0	0.000	321	18.5	90	6.2	0.000
% of those needing treatment on treatment	82	52.0	54	58.7	0.364	195	58.2	57	62.2	0.527
% of those on treatment with a controlled BP	24	28.4	18	36.4	0.359	99	53.4	20	35.6	0.026

*P*-value 0: comparing Intervention versus Control 1 at baseline.

*P*-value 1: comparing Intervention versus Control 1 at endline.

### Differences between both groups

Comparing the intervention and control group, we found no significant difference in the mean SBP reduction at population level (−0.32 mmHg, 95% CI −2.48 to 1.83) (Table 2). Likewise, no significant difference was detected in the DBP reduction between intervention and control (1.09 mmHg, 95% CI −0.29 to 2.46) (Table 2).

**Table 2:** Differences at population level (intervention, *n*=1,531; control, *n*=1,233)

	Estimate	95% CI		*p*
SBP (coefficient^a^)				
Difference between control and intervention areas at baseline	−1.46	−3.25	0.33	0.110
Difference between baseline and endline in intervention group	−2.75	−4.33	−1.18	0.001
Difference between baseline and endline in control group	−1.67	−3.17	−0.17	0.029
Difference-in-change between control and intervention	−0.32	−2.48	1.83	0.769
DBP (coefficient**a**)				
Difference between control and intervention areas at baseline	−2.68	−3.82	−1.54	0.000
Difference between baseline and endline in intervention group	−0.55	−1.54	0.44	0.278
Difference between baseline and endline in control group	−1.40	−2.37	−0.43	0.005
Difference-in-change between control and intervention	1.09	−0.29	2.46	0.121
Hypertension (odds ratio**b**)				
Difference between control and intervention areas at baseline	0.84	0.67	1.07	0.159
Difference between baseline and endline in intervention group	0.83	0.68	1.02	0.072
Difference between baseline and endline in control group	0.75	0.59	0.94	0.014
Difference-in-change between control and intervention	1.13	0.84	1.52	0.421
Tobacco use (odds ratio^b^)				
Difference between control and intervention areas at baseline	0.77	0.57	1.03	0.076
Difference between baseline and endline in intervention group	1.19	0.92	1.53	0.181
Difference between baseline and endline in control group	0.73	0.56	0.95	0.021
Difference-in-change between control and intervention	1.62	1.13	2.30	0.008
Alcohol use (odds ratio^b^)				
Difference between control and intervention areas at baseline	0.84	0.66	1.08	0.168
Difference between baseline and endline in intervention group	1.15	0.94	1.42	0.183
Difference between baseline and endline in control group	0.71	0.57	0.88	0.002
Difference-in-change between control and intervention	1.60	1.20	2.15	0.002
Insufficient fruit and vegetable consumption (odds ratio^b^)				
Difference between control and intervention areas at baseline	1.04	0.84	1.29	0.717
Difference between baseline and endline in intervention group	1.30	1.08	1.56	0.006
Difference between baseline and endline in control group	1.42	1.15	1.76	0.001
Difference-in-change between control and intervention	0.88	0.67	1.16	0.375
Inadequate physical activity (odds ratio^b^)				
Difference between control and intervention areas at baseline	0.54	0.24	1.19	0.127
Difference between baseline and endline in intervention group	1.58	0.87	2.88	0.135
Difference between baseline and endline in control group	8.14	4.63	14.31	0.000
Difference-in-change between control and intervention	0.20	0.09	0.45	0.000

a Coefficient are estimates from linear regression and represent the difference in the average of an outcome.

b Odds ratio are estimates from logistic regression and represent the difference in the likelihood of an outcome.

Among patients with hypertension at baseline, we found no significant difference in SBP reduction between intervention and control (−0.32 mmHg, 95% CI −5.15 to 4.52) (Table 3). However, DBP decreased more in the control hypertensive participants than among those in the intervention group (3.31 mmHg, 95% CI 0.36 to 6.26, *p=*0.028) (Table 3).

**Table 3:** Differences among hypertensives at baseline (intervention, n=388; control, n=266)

	Estimate	95% CI		*p*
SBP (coefficient^a^)				
Difference between control and intervention areas at baseline	1.66	−1.94	5.26	0.366
Difference between baseline and endline in intervention group	−14.82	−18.04	−11.61	0.000
Difference between baseline and endline in control group	−14.05	−17.71	−10.38	0.000
Difference-in-change between control and intervention	−0.32	−5.15	4.52	0.898
DBP (coefficient^a^)				
Difference between control and intervention areas at baseline	−0.69	−2.71	1.32	0.501
Difference between baseline and endline in intervention group	−7.55	−9.54	−5.57	0.000
Difference between baseline and endline in control group	−10.67	−12.89	−8.44	0.000
Difference-in-change between control and intervention	3.31	0.36	6.26	0.028
Awareness among hypertensives (odds ratio^b^)				
Difference between control and intervention areas at baseline	0.94	0.64	1.36	0.730
Difference between baseline and endline in intervention group	2.14	1.67	2.75	0.000
Difference between baseline and endline in control group	1.05	0.72	1.52	0.817
Difference-in-change between control and intervention	2.14	1.39	3.31	0.001
Drug treatment for hypertension (odds ratio^b^)				
Difference between control and intervention areas at baseline	0.59	0.21	1.67	0.323
Difference between baseline and endline in intervention group	0.81	0.42	1.56	0.529
Difference between baseline and endline in control group	0.64	0.20	2.03	0.452
Difference-in-change between control and intervention	1.84	0.57	5.94	0.306
Tobacco use (odds ratio^b^)				
Difference between control and intervention areas at baseline	0.64	0.34	1.18	0.152
Difference between baseline and endline in intervention group	0.99	0.58	1.69	0.974
Difference between baseline and endline in control group	0.51	0.28	0.90	0.021
Difference-in-change between control and intervention	1.91	0.88	4.12	0.101
Alcohol use (odds ratio^b^)				
Difference between control and intervention areas at baseline	0.66	0.39	1.13	0.132
Difference between baseline and endline in intervention group	0.94	0.59	1.48	0.778
Difference between baseline and endline in control group	0.62	0.38	0.99	0.044
Difference-in-change between control and intervention	1.39	0.73	2.64	0.309
Insufficient fruit and vegetable consumption (odds ratio^b^)				
Difference between control and intervention areas at baseline	0.90	0.56	1.44	0.657
Difference between baseline and endline in intervention group	1.41	0.95	2.11	0.092
Difference between baseline and endline in control group	1.39	0.88	2.20	0.153
Difference-in-change between control and intervention	1.01	0.55	1.83	0.979
Inadequate physical activity (odds ratio^b^)				
Difference between control and intervention areas at baseline	0.69	0.15	3.05	0.620
Difference between baseline and endline in intervention group	0.92	0.31	2.73	0.875
Difference between baseline and endline in control group	10.59	2.97	37.75	0.000
Difference-in-change between control and intervention	0.10	0.02	0.50	0.005

a Coefficient are estimates from linear regression and represent the difference in the average of an outcome.

b Odds ratio are estimates from logistic regression and represent the difference in the likelihood of an outcome.

We detected no difference between intervention and control populations in the reduction of hypertension prevalence (OR 1.13, 95% CI 0.84 to 1.52, *p=*0.421) but did find a significant improvement in the awareness of being hypertensive in the intervention population compared with the control population (OR 2.14, 95% CI 1.39 to 3.31, *p=*0.001).

With respect to behavioural and physiological risk factors, we found a significant decrease in the numbers of those reporting inadequate physical activity among the intervention group compared with the control group at population level (OR 0.20, 95% CI 0.09 to 0.45, *p<*0.001) and among the group with hypertension at baseline (OR 0.10, 95% CI 0.02 to 0.50, *p=*0.005). Alcohol use (OR 1.62, 95% CI 1.13 to 2.30, *p=*0.008) and tobacco use (OR 1.60, 95% CI 1.20 to 2.15, *p=*0.002) increased significantly in the intervention compared with the control group at population level.

### Outcomes within both groups

We found a significant reduction in mean SBP between baseline and endline measurements in both the intervention and control groups: 2.75 mmHg (95% CI 1.18 to 4.33) and 1.67 mmHg (95% CI 0.17 to 3.17), respectively (Table 2).

Among those with hypertension at baseline in the intervention (*n=*388) and control (*n=*266) settings, the reduction of SBP pre versus post was larger: 14.82 mmHg (95% CI 11.61 to 18.04) in the intervention and 14.05 (95% CI 10.38 to 17.71) at the control site. DBP decreased by 7.55 mmHg (95% CI 5.57 to 9.54 mmHg) in the intervention group and 10.67 mmHg (95% CI 8.44 to 12.89 mmHg) in the control group (Table 3).

In the control group, we also detected a decrease at population level in smoking (OR 0.73, 95% CI 0.56 to 0.95) and alcohol use (OR 0.71, 95% CI 0.57 to 0.88). Among patients with hypertension in the control group, smoking (OR 0.51, 95% CI 0.28 to 0.90, *p=*0.021) and alcohol use (OR 0.62, 95% CI 0.38 to 0.99, *p*=0.044) also reduced significantly. Insufficient intake of fruits and vegetables increased significantly at population level both in intervention (OR 1.30, 95% CI 1.08 to 1.56, *p=*0.006) and control (OR 1.42, 95% CI 1.15 to 1.76, *p=*0.001) settings.

## Discussion

In our study, we could not detect an effect attributable to a multi-component intervention composed of awareness campaigns, screening, and promotion of treatment and adherence in a slum setting compared with a control slum population. However, a significant decrease in BP was observed before and after the intervention period in the intervention and control groups, both at population level and among participants with hypertension at baseline. Furthermore, we found changes in cardiovascular risk factors with fruit and vegetable intake increasing in both groups, while tobacco and alcohol use diminished in the control group.

### Differences between both groups

Although we detected a decrease of BP among the participants in both groups, we found no additional significant difference between the intervention and control groups.

In all likelihood, we could not find an additional intervention effect because the above-mentioned intervention steps after the household screening were insufficient to yield an extra reduction in BP. Although we succeeded in doubling the rates of awareness in the intervention group from 10 to 21%, we were unable to successfully refer a sufficiently large group of people with hypertension to health care clinics and maintain them on hypertension medication (25) to see a significant effect compared with the control group. As described in the process evaluation paper of the SCALE-UP study (25), only a quarter of people diagnosed with hypertension were retained in care, and of those, only a third were able to achieve BP control. Therefore, among people with hypertension, as in the overall population, BP did not reduce significantly compared with the control group. Although participants in the intervention group could visit the local clinic twice a week, including the weekend, and get their medication for subsidized prices, they still reported that lack of time and money were the main reasons for them not showing up in the clinic for regular treatment resulting in poor adherence. It would be helpful to set up similar interventions where costs and waiting time for patients could be reduced through home-based management and medication for free, for example.

It might be suggested that the similarity in decrease of BP in the control group be caused by contamination through spill-over effects in the intervention community to the control community, despite the more than 8 km distance between the two slums. We checked that radio commercials run in Korogocho did not reach Viwandani, and although the slum population is very mobile, we have not come across study participants moving from Korogocho to Viwandani. It still could be that the health message from the campaigns has crossed the distance through friends and family of participants and that people at risk in the control study sought health care outside their slum. However, the clinics with subsidized medication and compliance programmes, stimulated through incentives for CHW, were strictly limited to Korogocho.

Our initial decision to develop and implement an integrated intervention focusing on awareness, access to screening and treatment, and adherence was based on earlier research showing that combination interventions have beneficial effects (11). Although health promotion was part of the intervention, as CHWs gave personal lifestyle advice, we did not put much emphasis on this as we thought it would be more feasible and cost-effective to concentrate on the delivery of care and treatment. However, health promotion might be more stimulated taking into regard the potential effect of a one-off screening and visit of a CHW in the control group. As awareness of hypertension is still very low in slum settings in Africa (6, 17, 27), a one-off measurement of BP and questionnaire might already have an effect on BP. The phenomenon of baseline measurements improving or modifying outcomes of participants has been described and has been seen in relation to BP (28, 29, Tanovic et al., unpublished). As research in the slums is very scarce, we were unable to locate studies specific to this setting.

Our results suggest that future interventions should concentrate on several steps in order to create a more sustainable reduction of cardiovascular risk. These would include increasing awareness and adoption of healthy lifestyles through screening and health promotion, ideally leading people with a relevant cardiovascular risk profile or hypertension to access the health care system, with consequent increased treatment and adherence levels.

### Outcomes within both groups

As BP levels are rising all over the continent (18), the reduction in SBP at population level in both intervention and control groups was substantial. The drop in SBP was even larger among people with hypertension of more than 14 mmHg. This is higher than other studies in SSA describing successful BP reduction, for example, through the introduction of health insurance to improve access to quality care (30).

As the reduction of BP was similar in both groups, it might be suggested that this was caused by an overall natural trend. It is possible, however, that neither Korogocho nor Viwandani had experienced any reduction in the prevalence of hypertension and related risk factors between 2008 and 2011 to support the idea of natural decrease prior to our study (APHRC, unpublished data). On the contrary, most studies in Africa have demonstrated significant increases in BP and related risk factors over relatively short time periods (18, 22).

A plausible explanation for the decrease in BP in both groups could be the effect of the baseline measurements of BP and questionnaires. Hypertensive participants in the control group might have sought treatment when they became aware of a high BP or adopted healthier lifestyles, as is shown in their significant reduction of tobacco and alcohol use, therefore improving their BP levels.

The increase in both groups of insufficient intake of fruits and vegetables and inadequate physical activity is in line with other studies in SSA (31) and is linked to the rapid growth in the numbers of overweight and obese people in these settings (32). However, the reduction in alcohol and tobacco use seen in the control group during the study period is contrary to expectations, given rising consumption trends in other slums (33). As the field workers and CHWs were trained together on CVD risk factors, it might be that some field workers in the control group, while conducting the interviews, provided some health advice that might have influenced behaviour regarding CVD risk factors in the control group.

### Strengths and limitations

The rapid growth of the urban poor in SSA and their increased CVD risk are a growing concern. The strengths of this study are that we were able to recruit a high number of participants in this challenging setting and that it is the first to describe outcomes of an intervention to reduce cardiovascular risk in slum settings in SSA.

Limitations might include that initially we had intended to measure blood glucose in order to determine the overall CVD risk; however, budgetary constraints and feasibility issues precluded this (25). As well, there were challenges during implementation (34), for example, to fully implement the support groups the way they were envisioned. This might have negatively influenced the results as the intended intervention was not fully operationalized. In addition, we acknowledge the existence of secular trends such as food price fluctuations and policy changes, which might have influenced our results, but were beyond the scope of our study.

## Conclusions

Although an effect attributable to a multi-component intervention could not be detected, reductions in BP were observed after the intervention period, in both intervention and control groups, on the population level as well as among participants with hypertension. Further research is needed to explore CVD prevention strategies focusing on screening and hypertension treatment adherence to effectively confront the increasing burden of CVD among Africa's urban poor. Although we aimed in our paper for a delivery of care model based on a literature review and expert opinions, we suggest to include in future studies the element of health promotion and population wide approaches, taking into account the potential impact on awareness and reduction of cardiovascular risk factors of a one-off screening among the adult population in low resource settings.
